# Agricultural Chemistry

**Published:** 1856-01

**Authors:** 


### AGRICULTURAL CHEMISTRY.

During this session, arrangements have been made so that a course of lectures on this department will be given by Prof. Marsh, our present accomplished Professor of Chemistry and Materia Medica, and notice of the fact was given through the columns of the secular newspapers. How general this notice has been made, we have not had the means of knowing, but inasmuch as it was intended as a public benefit, in which that largest and first industrial interest of our country is particularly interested, we hoped they would certainly be informed of it by those who are ever ready to advise them of every thing touching their well-being.

As this institution is a State enterprise, we believed it to be our duty to institute this course of lectures, which if we are convinced it is desirable on the part of the farming class of our fellow-citizens, it will be in future a permanent arrangement. We therefore respectfully call especial attention to this notice, and would be glad to have an expression of opinion in the premises.

The above course will commence on the 20th. The fee is nominal, amounting only to the usual charge of matriculation, which is three dollars only.

